# An Autopsy Case of Fulminant Systemic Infection of *Clostridium perfringens* With a Diverse Role of Toxins in a Healthy Patient

**DOI:** 10.1155/2024/9213132

**Published:** 2024-09-14

**Authors:** Ayano Osamura, Hiromi Onizuka, Kenta Masui, Kumiko Murakami, Tomoko Yamamoto, Yoji Nagashima, Munekazu Takeda, Atsushi Kurata

**Affiliations:** ^1^ Department of Pathology Tokyo Women's Medical University, Shinjuku, Tokyo 162-8666, Japan; ^2^ Department of Pathology Kyorin University, Mitaka, Tokyo 181-8611, Japan; ^3^ Department of Surgical Pathology Tokyo Women's Medical University, Shinjuku, Tokyo 162-8666, Japan; ^4^ Department of Critical Care and Emergency Medicine Tokyo Women's Medical University, Shinjuku, Tokyo 162-8666, Japan

## Abstract

We herein report an autopsy case of a fulminant *Clostridium perfringens* (*C. perfringens* or Welch bacilli) infection in a healthy adult. A 72-year-old, immunocompetent man visited the emergency department with lower back pain, and blood test revealed hemolytic attack. His condition rapidly worsened with severe acidosis and anemia, and he died despite symptomatic treatment. An autopsy examination demonstrated an abscess with necrosis and air spaces in the right lobe of his liver. Numerous Gram-positive bacilli were seen in the liver and bone marrow, and *C. perfringens* was identified in culture of the antemortem blood sample. Of note, a mucosal epithelium of the ileum showed loss of tight junctions (claudin 4), suggesting the involvement of *C. perfringens* toxins with its systemic spreading. Welch toxins were suggested to be involved in serious pathological conditions such as hemolytic anemia and systemic infections, and it is necessary to raise Welch infection as one of the differential diagnoses for fulminant systemic infections even in healthy individuals.

## 1. Introduction

*Clostridium perfringens* (*C. perfringens*), also known as Welch bacilli, is one of the resident microbes in the large intestines of humans and animals. *C. perfringens*, a Gram-positive anaerobic rod, is subclassified into five toxinotypes (A, B, C, D, and E) according to the combination of its major toxins including alpha (CPA), beta (CPB), epsilon (ETX), and iota (ITX) [1]. Further, the bacteria could produce other lethal toxins such as enterotoxin (CPE), perfringolysin O (PFO), and beta2 toxin (CPB2) [2]. The pathogenesis of *C. perfringens* infection is mediated by one or more of these toxins (e.g., *α*-toxin causes intravascular hemolysis while CPE could damage enteric walls) [1], and the bacteria accordingly cause human infectious diseases such as food poisoning, gas gangrene, or purulent infection. Sepsis is rare and more likely to occur in immunocompromised hosts [3], and once sepsis develops, severe intravascular hemolysis and multiple organ failure cause death of the patients within a short period of time [4]. Conversely, fulminant infection of *C. perfringens* is extremely rare in an immunocompetent healthy person. Here, we report a rare autopsy case of fulminant *C. perfringens* infection in an immunocompetent elderly, suggesting that diverse roles of its toxins could contribute to an aggressive disease course in a healthy adult patient.

## 2. Case Presentation

A 72-year-old male with a past history of cholecystitis, glaucoma, and cerebral aneurysm had been engaged in weeding 2 days before admission. He did not possess any scratch in his skin but developed lower back pain. His symptoms did not improve, and he was admitted to a hospital because blood tests suggested a hemolytic attack, hepatorenal dysfunction, and disseminated intravascular coagulation (DIC) (Table 1). At the time of admission, the patient was placed on a ventilator due to his poor oxygenation in the blood. He showed a strong tendency to bleed, and cardiac arrest occurred 10 min later. Despite the treatment of severe acidosis and anemia with sodium bicarbonate, blood transfusion, and administration of albumin, he did not recover and died.

**Figure 1:**
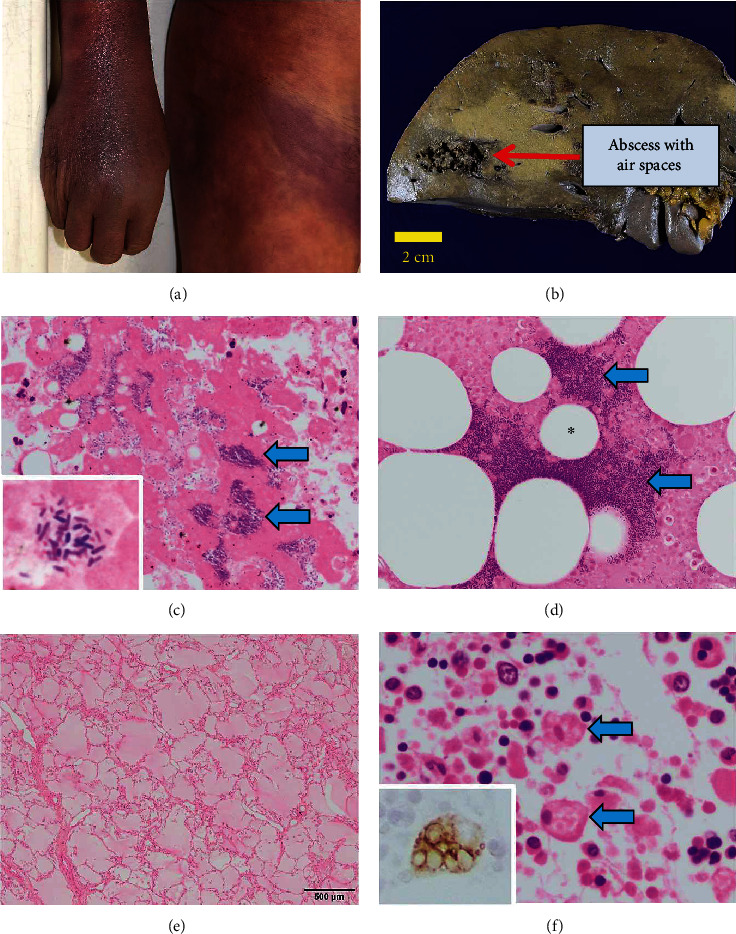
Autopsy findings of fulminant systemic *C. perfringens* infection in a healthy patient. (a) The skin and soft tissue of the upper extremities showed swelling with gas and subcutaneous hemorrhage. (b) There was abscess formation with multiple air spaces in the right lobe of the icteric liver. Scale bar, 2 cm. (c) Hematoxylin and eosin (HE) section of the liver tissue showed coagulative necrosis of the hepatocytes with numerous bacterial colonies (arrows). Inset, magnified view of the bacterial colony (Gram-positive rods) with Gram staining. (d) HE section of the bone marrow from the lumber spine showed coagulative necrosis of the marrow tissue with massive bacterial colonies (arrows) and multiple bubbles (asterisk). (e) Both lungs displayed congestive edema (HE staining). (f) Hemophagocytic activities of the macrophages (arrows) were significantly increased in the bone marrow (HE staining). Inset, magnified view of CD68-positive hemophagocytic macrophages.

Subsequently, autopsy was performed after 15.5 h of his death to determine the cause of death in this patient. His trunk and extremities were swollen with wide-spreading subcutaneous hemorrhage (Figure 1(a)). Abscess formation with necrosis and air spaces was observed in the right lobe of the liver (1300 g) (Figure 1(b)). Numerous Gram-positive bacilli were seen under the microscope (Figure 1(c)), and *C. perfringens* was identified in culture from the antemortem blood sample. Many rods were also found in the vertebral marrow (Figure 1(d)), indicating a systemic distribution of the pathogens. We considered the findings of swollen extremities and necrosis with air spaces in the liver and bone marrow as the ones related to gas gangrene. Consistent with the status of systemic inflammatory response syndrome (SIRS), there were congestive pulmonary edema (left 780 g and right 1100 g), centrilobular necrosis of the liver with reactive hepatitis, acute splenitis (185 g), shock kidney (tubular degenerative necrosis) (left 240 g and right 210 g), and hemophagocytosis in each organ of the patient (Figures 1(e) and 1(f)). Based on the pathological findings, we concluded that the patient died due to septic shock caused by *C. perfringens* systemic infection.

**Figure 2:**
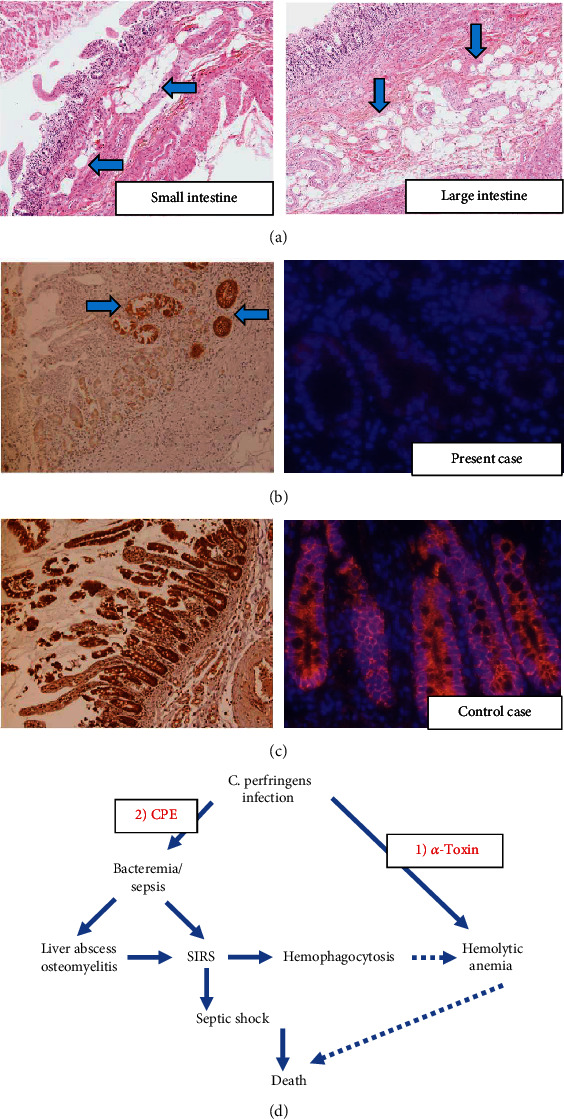
A diverse role of *C. perfringens* toxins in the pathogenesis of fulminant infection in a healthy patient. (a) HE section of ileum (left panel) and ascending colon (right panel) showed multiple bubbles in the intestinal walls (arrows). Immunostaining of tight junction–related molecule claudin 4 showed massive loss of its reactivity in the ileal epithelium of the present case (b), in comparison with positive control (autopsied ileal samples from non-Welch infection patient) (c). Note that some intact glands preserved the immunopositivity of claudin 4 as an internal control in the present case (arrows) (b). Immunostaining protocol was according to the previous reports [7]. Left panels, stained with 3,3′-diaminobenzidine (DAB) (brown, claudin 4); right panels, Alexa Fluor 555–stained immunofluorescence (red, claudin 4; blue, 4′,6-diamidino-2-phenylindole (DAPI)). (d) A diverse role of *C. perfringens* toxins in the pathogenesis of fulminant infection of the present case. (1) *α*-toxin could induce intravascular hemolytic attack, and (2) CPE could destruct claudin 4 of tight junctions and facilitate systemic dissemination of the bacilli even in this immunocompetent host. CPE, *C. perfringens* enterotoxin; SIRS, systemic inflammatory response syndrome. Solid arrows indicate deterministic causality; dotted arrows indicate presumptive causality.

We took further examinations to formulate the possible pathogenic mechanism to contribute to an aggressive clinical course of the patient. Recent reports indicated that the toxin *Clostridium perfringens* enterotoxin (CPE) produced by Welch bacilli could destroy claudin molecules (claudin 4) that maintain tight junctions of the intestinal epithelial cells, potentially promoting systemic dissemination of the bacilli [5, 6]. Of interest, mild pneumatosis cystoides intestinalis–like air spaces were observed in the small and large intestines (Figures 2(a)), and immunostaining of claudin 4 (clone EPRR17575, Abcam, Cambridge, UK) showed massive loss of its reactivity in the mucosal epithelium of his lower gastrointestinal tract (positive control: autopsied intestinal samples from non-Welch infection patient) (Figures 2(b) and 2(c)), suggesting destruction of enterocytes' tight junctions and involvement of peculiar toxins to the pathogenesis of the fulminant infection in this immunocompetent host (Figure 2(d)).

## 3. Discussion

*C. perfringens* or Welch bacilli could cause severe symptoms, especially in the hosts with several underlying conditions and risk factors such as malignant tumors, diabetes, liver cirrhosis, and blood disorders as well as undergoing treatments with surgery, chemotherapy, and steroid [8]. Conversely, this case had no specific history which could weaken his immunity status. It is thus considered that this is a very rare case that systemic fulminant *C. perfringens* infection occurred in a healthy adult, and it should be recognized as one of the differential diagnoses for fulminant systemic infections even in otherwise healthy people.

Pathogenic factors of the severe condition in this patient were potentially based upon intravascular hemolytic reaction as well as circular distribution of the pathogens. Various toxins are known to contribute to the pathogenesis of Welch bacilli, and one of such toxins is the main toxin produced by *C. perfringens*, *α*-toxin. It is a lecithinase with phospholipase C activity that degrades phospholipids [9]. The lecithinase acts on phospholipids of the cell membrane, damaging red blood cell membrane to induce hemolysis [1]. Furthermore, it is known that in the microcirculatory system, vascular endothelial damage causes platelet aggregation, and DIC is subsequently induced as a result of peripheral circulation damage and thrombus formation. Considering the severe hemolytic attack of the patient, *α*-toxin could play a major role in this patient's aggressive clinical course from its early phase (Figure 2(d)).

Additionally, it has been reported that the toxin CPE could help the bacilli to access the circulatory system by destructing claudin 4 that constitutes tight junctions of the enterocytes [5, 6]. We thus examined the status of claudin 4 in the patient's sample, based on the hypothesis that destruction of claudin 4 may be involved in pathogen invasion and fulminant disease. Comparing the intestinal epithelial cells of healthy individuals, more claudin molecules were shed in the enterocytes of the case. Generally, Welch bacilli are considered to invade the systemic circulation from the damaged part of the gastrointestinal mucosa, especially in the immunocompromised or intensively treated patients [10]. In healthy individuals, intestinal defense mechanisms are basically intact and prohibits bacteria from systemically spreading. Although initial access/colonization of the bacilli in the host was unclear from the skin or gut, our case poses a caveat that Welch bacilli could enter the systemic circulation by destructing the tight junction of the intestine even in an immunocompetent healthy patient (Figure 2(d)).

In summary, we report an autopsy case of a fulminant *C. perfringens* (Welch bacilli) infection in a healthy adult, including a discussion on the contribution of pathogen's toxin to its pathogenesis. Welch toxins were suggested to be involved in serious pathological conditions such as hemolytic anemia and systemic infections even in healthy individuals, which could be a potential therapeutic target [11]. A potential of this rare systemic infection should be thus suspected from the symptomatic combination of sepsis with hemolytic attack, and immediate intensive treatment should be adopted against it.

## Figures and Tables

**Table 1:** Laboratory data at the time of admission of the present case.

**Blood test**	
WBC (10^3^/*μ*L)	5.04
RBC (10^6^/*μ*L)	N.D. (hemolysis)
Hb (g/dL)	5.5
Plt (10^4^/*μ*L)	N.D.
PT (s)	> 200.0
APTT (s)	> 150.0
FDP (*μ*g/mL)	> 200.0
D-dimer (*μ*g/mL)	> 100.0
Fibrinogen (mg/dL)	< 50
CRP (mg/dL)	3.94
AST (U/L)	1300
ALT (U/L)	348
LDH (U/L)	9900
BUN (mg/dL)	39.0
Cre (mg/dL)	2.19
Na (mmol/L)	135
K (mmol/L)	6.8
Lactate (mmol/L)	14.3
pH	6.922
PO_2_ (mmHg)	410.4
PCO_2_ (mmHg)	19.1
BE (mmol/L)	−26.2
HCO_3_ (mmol/L)	3.8

*Note:* The blood test showed severe anemia, inflammation, liver and kidney dysfunction, metabolic acidosis, and DIC.

Abbreviation: N.D., not detected.

## Data Availability

All data is contained in the manuscript.
